# CRABP2 (Cellular Retinoic Acid Binding Protein 2D): A novel biomarker for the diagnosis and prognosis involved in immune infiltration of lung adenocarcinoma

**DOI:** 10.7150/jca.96518

**Published:** 2025-02-03

**Authors:** DaXia Cai, Feng Tian, DengKe Zhang, JianFei Tu, YongHui Wang

**Affiliations:** 1 Cancer Center, Lishui Central Hospital, The Fifth Affiliated Hospital of Wenzhou Medical University, Lishui Hospital of Zhejiang University, Lishui 323000, China.; 2 Department of Stomach Enterochirurgia, Lishui People's Hospital, the Six Affiliated Hospital of Wenzhou Medical University, Lishui, Zhejiang, China.; 3 Department of Pulmonary and Critical Care Medicine, The First Affiliated Hospital of Jinan University, Guangzhou, Guangdong, China.

**Keywords:** CRABP2(Cellular Retinoic Acid Binding Protein 2), Biomarker, LUAD (lung adenocarcinoma), diagnosis, cell cycle, apoptosis, Immune checkpoint

## Abstract

**Objective:** Overexpressed CRABP2 (Cellula Retinoi Aci Bindin Protei 2D) can promote progression of various tumors. However, there are few comprehensive analysis studies on CRABP2 in lung adenocarcinoma (LUAD).

**Methods:** Several large public databases and online analysis tools such as TCGA, GEO, GEPIA2, UALCAN, Kaplan Meier plotter, LinkedOmics, TIMER, CCLE and Metascape were used for big data mining analysis. RNA interference technology, CCK8 assay, flow cytometry and apoptosis detection, and western blot were used for *in vitro* experiments.

**Results:** The study revealed that the expression level of CRABP2 in plasma were higher (mean level 31.6587 ±13.8541 ng/mL vs. 13.9328 ± 5.5805 ng/mL, *p*<0.0001) in patients with early stage (stage IA) LUAD compared to the control group based on analysis of 640 LUAD patients and 640 matched healthy control plasma samples from Lishui Central Hospital. Receiver Operating Characteristic curve showed that CRABP2 had certain accuracy in predicting early LUAD, with a sensitivity of 70.98%, a specificity of 94.53%, a cut-off value of 0.6551 ng/mL, and an Area Under the Curve of 0.839 (95%CI: 0.817 - 0.859, *p*<0.0001). Compared with normal lung tissue, CRABP2 was significantly overexpressed in LUAD (*p*<0.05). High CRABP2 expression in LUAD predicts poor prognosis both in Overall Survival (95%CI: 1.04-1.46, HR:1.23, *p*=0.018) and FP (First Progression, 95%CI: 1.10-1.65, HR = 1.35, *p*=0.0032) in LUAD patients. CRABP2 can promote the progression of LUAD by promoting the G2/M phase transition, inhibiting the apoptosis and participating in the regulation of immune microenvironment. The high expression of CRABP2 will inhibit the recruitment of immune effector cells and promote the proportion of immuno-suppressive cells, thus promoting the progression of LUAD. The low expression of CRABP2 may enhance the expression of CD274(PD-L1), HAVCR2 and PDCD1LG2(PD-L2) in LUAD. While, the high expression of CRABP2 may enhance the expression of CTLA4, LAG3, PDCD1(PD-1), TIGIT and IGSF8 in LUAD.

**Conclusions:** CRABP2 may be a valuable biomarker for diagnosis, treatment and prognosis of LUAD. Patients with high expression of CRABP2 in LUAD may have suboptimal efficacy when treated with inhibitors targeting CD274, HAVCR2, and PDCD1LG2, whereas they may experience better efficacy with inhibitors targeting CTLA4, LAG3, PDCD1, TIGIT, and IGSF8. Most of cancer patients with high CRABP2 expression may benefit from immune checkpoint inhibitor therapy. Our study results have laid a positive foundation for LUAD diagnosis and therapy.

## Introduction

Lung cancer remains the leading cause of cancer-related mortality and incidence globally, with a disheartening 5-year survival rate that hovers below 20% 1. Lung adenocarcinoma (LUAD), the predominant histopathological subtype of non-small cell lung cancer (NSCLC), is particularly challenging due to its lack of distinctive clinical features in the early stages, which hampers early detection 2. The prognosis for patients diagnosed with LUAD is grim, with a markedly low 5-year survival rate post-diagnosis 3. Hence, the imperative for early detection in LUAD cannot be overstated, as it is pivotal in curtailing lung cancer mortality. And current therapeutic strategies for LUAD encompass a spectrum of treatments, including chemotherapy, targeted therapy, surgery, immunotherapy, radiation, and combinations thereof. The last decade has witnessed a transformative era in cancer therapy with the emergence of targeted and immunotherapies, especially the introduction of immune checkpoint inhibitors that have demonstrated remarkable efficacy 4. Despite these advancements, a significant portion of LUAD patients exhibit resistance to these novel treatments. Moreover, in the absence of effective therapeutic targets within key oncogenic signaling pathways, many LUAD patients are left without targeted treatment options 5,6. Therefore, it is urgent to identify novel biomarkers for early clinical diagnosis, effectively therapeutic targets, and effective biomarkers for immunotherapy response in LUAD. Encouragingly, several PD-L1 and PD-1 targeting drugs have received approval from the United States Food and Drug Administration (FDA) for the treatment of LUAD 7. Landmark clinical trials such as KEYNOTE 189, CheckMate 9LA, KEYNOTE 407, and KeyNOTE-021G have demonstrated that the survival of patients with advanced LUAD can be significantly enhanced with combination regimens centered on PD-1 immune checkpoint inhibitors, which have since been FDA-approved 8-11. Therefore, further exploration of immune-related molecular regulatory networks and biomarkers is critical and may offer a foundation for devising treatment strategies and prognostication in LUAD. The pursuit of deeper understanding could pave the way for more personalized and efficacious therapies for patients afflicted with this devastating LUAD.

Cellular Retinoic Acid Binding Protein 2 (CRABP2), a member of the intracellular lipid-binding protein family, plays a crucial role in the cellular metabolism of vitamin A. It facilitates the transport of all-trans-retinoic acid (RA), a metabolite of vitamin A, to the retinoic acid receptor (RAR) located in the cell nucleus, thereby influencing key cellular processes such as metastasis, proliferation, invasion, and apoptosis 12. Emerging evidence suggests a correlation between the dysregulation of CRABP2 expression and the development of various human cancers. Notably, it has been implicated in promoting the invasive and metastatic capabilities of cancers, including pancreatic 13,14, bladder, and head and neck squamous cell carcinomas 15. Interestingly, CRABP2 has also been observed to exert inhibitory effects on breast cancer progression through distinct mechanisms 16. Furthermore, the downregulation of CRABP2 has been shown to induce apoptosis and impede the metastatic spread in esophageal squamous cell carcinoma 17. Its aberrant expression has been linked to adverse outcomes in malignant gliomas 18. In a phase I clinical study, CRABP2 expression emerged as a potential biomarker for predicting the therapeutic response to gemcitabine-nab-paclitaxel in pancreatic cancer 13. Despite these insights, the precise function and underlying mechanisms of CRABP2 in LUAD, as well as its potential as a diagnostic and prognostic indicator, remain to be elucidated. Further research is warranted to fully understand the multifaceted role of CRABP2 in LUAD and to harness its potential in advancing our therapeutic strategies against this aggressive malignancy.

In our research, we conducted a comprehensive analysis by collecting plasma samples from patients with lung adenocarcinoma (LUAD) and quantified the levels of Cellular Retinoic Acid Binding Protein 2 (CRABP2). We then assessed the correlation between CRABP2 expression and the prognosis of LUAD patients using extensive public databases. Our investigation extended to exploring the potential mechanisms of CRABP2 action in LUAD by leveraging two robust databases: LinkedOmics and Metascape. Our findings suggest that CRABP2 may facilitate the advancement of LUAD by modulating the tumor cell cycle and impeding apoptosis in LUAD cells. To substantiate our discoveries in big data, we experimentally validated the hypothesis by establishing a CRABP2 knockdown LUAD cell line *in vitro*. This approach allowed us to directly observe the effects of CRABP2 in LUAD. Furthermore, we delved into the intricate relationship between CRABP2 and the tumor immune microenvironment, immune regulation, and immune checkpoint pathways. Our results revealed a significant negative correlation between CRABP2 expression and the expression of immune checkpoint-related genes such as CD274, PDCD1LG2, and HAVCR2 in LUAD, while a positive correlation was observed with PDCD1, CTLA4, TIGIT, and LAG3. This study elucidates the potential mechanisms of CRABP2's influence on tumor-immunity in LUAD, offering valuable insights that could pave the way for early diagnosis, precise therapeutic targeting, and immunotherapy strategies of LUAD. Collectively, our research provides a strong theoretical basis for improving the prognosis and survival of LUAD patients.

## Materials and Methods

### Determination of CRABP2 levels in plasma samples

Stored plasma was collected from Lishui Central Hospital from June 2018 to June 2023, and the detailed inclusion criteria for patient selection are as follows: a) Undergoing surgery with pathological confirmation of LUAD; b)Patients with stage IA were confirmed according to the 8th edition of the AJCC(American Joint Committee on Cancer) TNM (Tumor Node Metastasis) staging system; c)No history of autoimmune diseases, interstitial lung diseases, asthma, or chronic obstructive pulmonary disease; d) No infection in the last 6 months (including bacterial infection, viral infection, mold infection, tuberculosis infection, etc.); e) Patients who have not received anti-tumor therapy; f) No history of malignancies; g)No history of other cancers; h) Obtain written informed consent from the patient or the patient's family; i) Pathological specimen after radical surgical treatment; j) The patient was at least 18 years old at the time of diagnosis. Based on the above inclusion criteria, 640 patients were included in this study, and 640 matched healthy control plasma samples were obtained from individuals free of any known malignancy or chronic lung disease. We collected blood samples before the patient's initial diagnosis and treatment. The blood samples were separated using a centrifuge at 2500 rpm for 20 minutes, which was stored at -80℃ in the Key Laboratory of Imaging Diagnosis and Minimally Invasive Research, Lishui Central Hospital. Clinical data of LUAD patients and controls were collected including tumor histological type, comorbidities, disease stage, and overall survival time. Expression of CRABP2 in plasma was detected using ELISA (Enzyme Linked Immunosorbent Assay) according to the manufacturer's instructions. Prepare a series of dilution standards ranging from 20 ng/mL to 0 (0, 0.312, 0.625, 1.25, 2.5, 5, 10, and 20 ng/mL) in 10 mM phosphate-buffered saline. All samples in our study were tested in duplicate, as the previous operation 19.

### Databases and online analysis tools

The GEO (Gene Expression Omnibus, https://www.ncbi.nlm.nih.gov/geo/) and GEPIA2 (Gene Expression Profiling Interactive Analysis, http://gepia2.cancer-pku.cn/#index) databases were used to analyze the differential expression of CRABP2 in LUAD and normal lung cancer tissues at the mRNA level. Differential analysis of total protein expression of CRABP2 was performed using UALCAN 19 database. Only patients with no history of cancer other than lung cancer were included in the study. Expression microarray series of CRABP2 from the GEO dataset including GSE10072 (GPL96), GSE31210 (GPL570) 20, GSE31908 (GPL96), GSE32863 (GPL6884) 21, and GSE116959 (GPL17077) 22, which containing LUAD tumor and non-tumor samples, and the details of 6 GEO datasets were summarized in Table 2.

The Kaplan Meier plotter 23 was utilized to evaluate the correlation between the expression of CRABP2 and survival rates in a large number of LUAD samples sourced from the GEO and TCGA databases. LinkedOmics 24 (http://www.linkedomics.org) was employed to examine the differential expression of CRABP2 in LUAD. The TIMER database 25 (https://cistrome.shinyapps.io/timer/) was used for a comprehensive analysis of tumor-infiltrating immune cells across various cancer types. We obtained data on CRABP2 expression in common human LUAD cell lines from the CCLE (Cancer Cell Line Encyclopedia, https://sites.broadinstitute.org/ccle) database. Additionally, we downloaded RNA-sequencing expression profiles and clinical information of CRABP2 from the TCGA dataset (https://portal.gdc.com) to evaluate the integration of six state-of-the-art algorithms for immune score assessment, including TIMER, EPIC, MCP-counter, QUANTISEQ, XCELL, and CIBERSORT.

### Cell cultures and transfections

The LUAD cell lines, NCI-H1650 and A549, were procured from ATCC (American Type Culture Collection; located in Manassas, VA, USA). These cell lines were maintained in an incubator set at 37°C and 5% CO2. For their cultivation, Dulbecco's modified Eagle's medium (DMEM) was used, fortified with 100 U/mL penicillin, 100 µg/mL streptomycin (sourced from HyClone, USA), and 10% fetal bovine serum (FBS; supplied by Gibco, Carlsbad, USA).

The siRNA s (mall interfering RNA) was bought from Genepharma (Shanghai, China) to knock down CRABP2. The Target sequences of CRABP2 were siCRABP2 a, 5'-AGGAGGGAGACACTTTCTACA-3'; siCRABP2 b, 5'-CTGTAGCCTATACAGTTTAGA-3'; and siCRABP2 c, 5'-GCACCACAGAGAUUAACUUTT-3'. Negative control (NC) sequence: 5′-UUCUCCGAACGUGUCACGUTT-3′. NCI-H1650 and A549 cells were transfected with Lipofectamine™ 3000 Transfection Reagent (Thermo Fisher Scientific, Waltham, USA).

### Western blotting analysis, cell cycle/apoptosis assay and cell proliferation assay

Western blotting, cell cycle/apoptosis assay and cell proliferation assay were conducted according to the methods outlined in a previous study 26. The antibodies employed included CRABP2 antibody (1:1,000 dilution, sourced from ProteinTech, Cat. # 10225-1-AP), anti-human β-Actin (1:1000 dilution, #4970 from Cell Signaling Technology, Danvers, MA, USA), and anti-rabbit IgG (1:1000 dilution; #7074 from Cell Signaling Technology). For the cell proliferation assay, we utilized the Cell Counting Kit 8 (CCK-8) assay (supplied by Beijing Solar Science & Technology Co, Beijing, China). Additionally, we used cell apoptosis staining solution (AP101; sourced from Multisciences (Lianke) Biotech Co., Ltd.) and cell cycle staining solution (CCS012) also from Multisciences (Lianke) Biotech Co., Ltd., Hangzhou, China] for the cell apoptosis and cell cycle assays, respectively. Cell suspensions were evaluated using Flow cytometry (ACEA NovoCyte, provided by Agilent Technologies, Santa Clara, USA). All experiments were replicated three times to ensure reliability.

### Ethics statement

The study protocol was approved by the Ethics Committee of Lishui Central Hospital. All subjects signed informed consent at the time of enrollment.

### Statistical methods

Student's t-test or Wilcoxon rank-sum test were used in evaluating our continuous variables. Statistical analysis and visualization were performed by GraphPad Prism software version 8.0. Data related to this study can be accessed from the corresponding authors upon a reasonable request. *p*<0.05 was used to assess differences. ^#^*p*>0.05, ^*^p<0.05, ^**^p<0.01, ^***^p<0.001, ^****^p<0.0001, asterisks (^*^) stand for significance levels.

## Results

### CRABP2 was significantly overexpressed in plasma of LUAD patients

In this study, plasma samples from 640 LUAD patients diagnosed with TNM stage IA and 640 samples from healthy volunteers were analyzed. As shown in Table 1, the mean age of the LUAD patients was 56.1 years, and the number of cigarette smoking was 80. Female patients predominated (70.6%, 452 patients) in LUAD groups. There were significant differences between LUAD patients and controls with regard to age, sex, with diabetes, hypertension, chronic liver disease, chronic airway disease, and history of other cancers (Table 1). The majority of LUAD patients had stage IA1.

**Figure 1:**
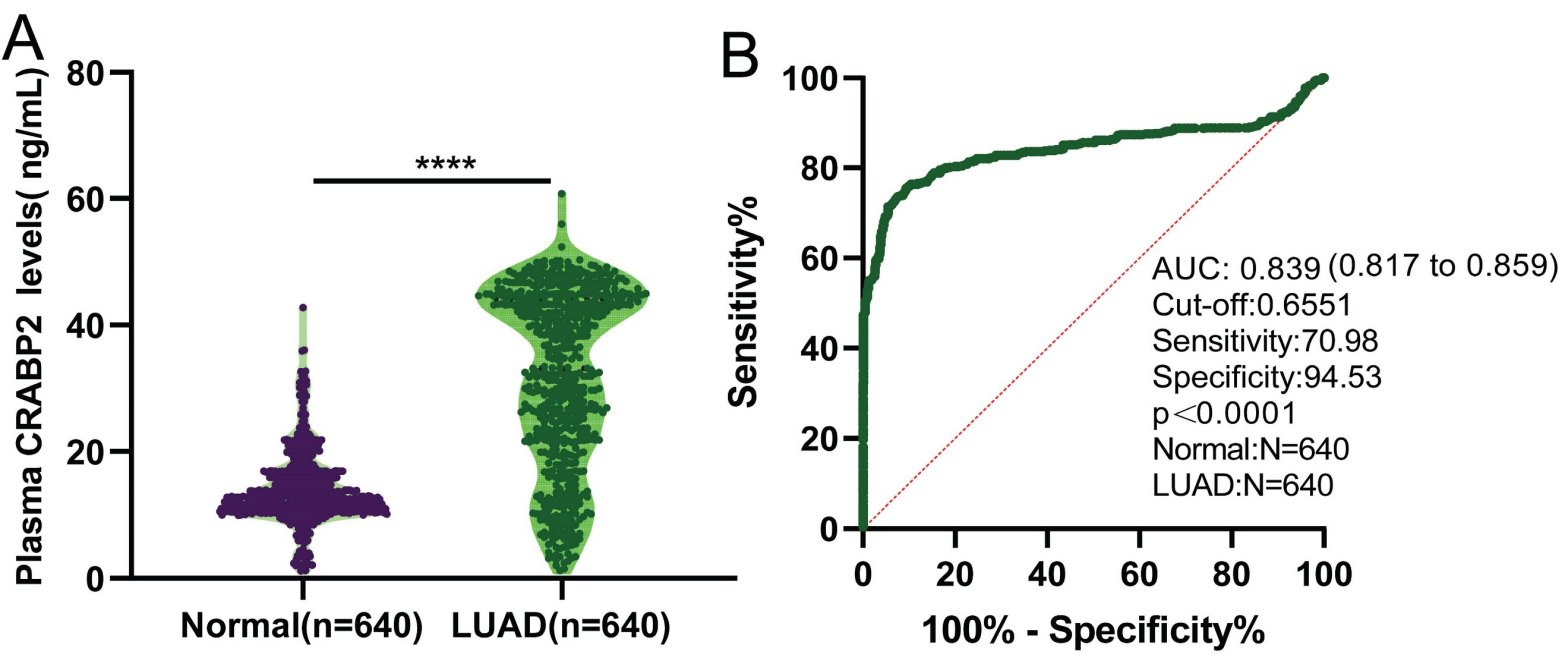
Plasma CRABP2 levels and ROC curve for plasma CRABP2 LUAD patients at stage IA and controls. (A) Plasma CRABP2 levels in specimens from LUAD patients at stage IA and controls. (B) ROC curve for plasma CRABP2 in LUAD patients at stage IA. CRABP2: cellular retinoic acid binding protein 2; ROC: receiver operator characteristic; AUC: area under the curve; LUAD: lung adenocarcinoma. ^****^p<0.0001, asterisks (^*^) stand for significance levels. *p*<0.05 was used to assess differences.

Our findings revealed a notable elevation in the expression levels of CRABP2 among LUAD patients in stage IA compared to the plasma of healthy individuals (Figure 1A, mean level 31.6587 ±13.8541 ng/mL vs. 13.9328±5.5805 ng/mL, *p*<0.0001). Utilizing the Receiver Operating Characteristic (ROC) curve, we established a threshold value for plasma CRABP2 levels, enabling us to differentiate between LUAD patients in the TNM stage IA group and the control group (Figure 1B). The diagnostic efficiency proved to be remarkably high, exhibiting a sensitivity of 70.98%, a specificity of 94.53%, a cut-off value of 0.6551 ng/mL, and an AUC (Area Under the Curve) of 0.839 (95%CI: 0.817 - 0.859; Figure 1B, *p*<0.0001). These results imply that the expression of CRABP2 in plasma could potentially serve as a sensitive biomarker for diagnosing LUAD patients in stage IA and might even be considered as a precise therapeutic target for LUAD.

### CRABP2 was overexpressed in LUAD compared with normal lung tissue

**Figure 2:**
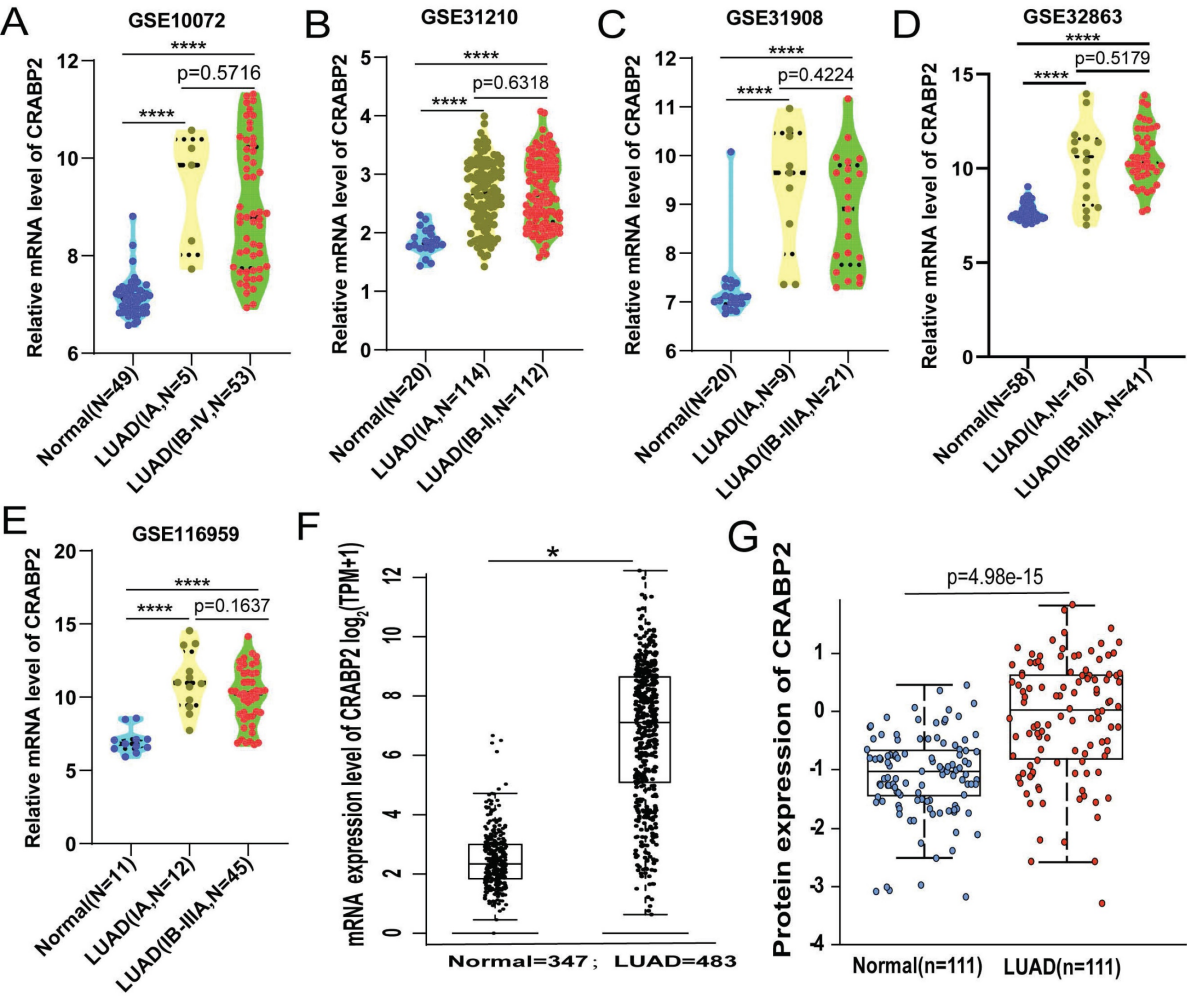
The different expression of CRABP2 in LUAD. (A-E) CRABP2 mRNA expression comparisons between normal and LUAD tissues obtained from the GEO database (Student T test). (F) Comparison of CRABP2 mRNA expression between normal and LUAD tissues in TCGA-LUAD cohort through GEPIA2 online database (unpaired Wilcoxon test). (G) CRABP2 protein expression comparison between normal and tumor tissues obtained from the UALCAN web tool (Wilcoxon test). TCGA: The Cancer Genome Atlas; LUAD: lung adenocarcinoma; *p*-value<0.05 was used to assess differences. ^*^p<0.05, ^**^p<0.01, ^***^p<0.001, ^****^p<0.0001, asterisks (*) stand for significance levels.

As of now, several large-scale clinical studies 27-29 have not included stage IA in the scope of postoperative adjuvant therapy, while LUAD from stage IB to stage III has been included in the scope of postoperative adjuvant therapy. Therefore, we consider stage IA LUAD to represent a more early-stage LUAD population. Consequently, we downloaded mRNA expression data for CRABP2 in LUAD and normal lung tissues from the GEO database, divided into two subgroups: patients with stage IA LUAD and those with LUAD from stage IB and beyond. The results revealed that, compared to normal lung tissues, the expression level of CRABP2 significantly increased in stage IA. Interestingly, the expression level of CRABP2 also showed a significant increase in those with LUAD from stage IB and beyond, however, there was no significant difference between the two subgroups (patients with stage IA LUAD and those with LUAD from stage IB and beyond) (Figure 2A-E).

Furthermore, we have downloaded mRNA data for CRABP2 from both LUAD and normal lung tissues from the GEO database, in preparation for conducting a ROC curve analysis. Our analysis revealed that the expression of CRABP2 demonstrates relatively high diagnostic accuracy, exhibiting high sensitivity, specificity, and a distinct cut-off value in middle and late-stage LUAD (Supplementary Material S1, *p*<0.0001).

Moreover, we checked TCGA-LUAD cohort through GEPIA2 online database, and patients with LUAD encompassing all stages, from Stage I to Stage IV, showing that CRABP2 was significantly overexpressed in all stages of LUAD (*p*<0.05, Figure 2F). The expression of CRABP2 protein was checked in UALCAN database, where it was found that it is highly expressed in all stages of LUAD, and consistent with the above (*p*<0.05, Figure 2G).

The above results suggests that expression of CRABP2 might be sensitive biomarker for diagnosis of more early-stage LUAD population, and could potentially serve as precise therapeutic targets for LUAD in early, middle and late stage.

**Figure 3:**
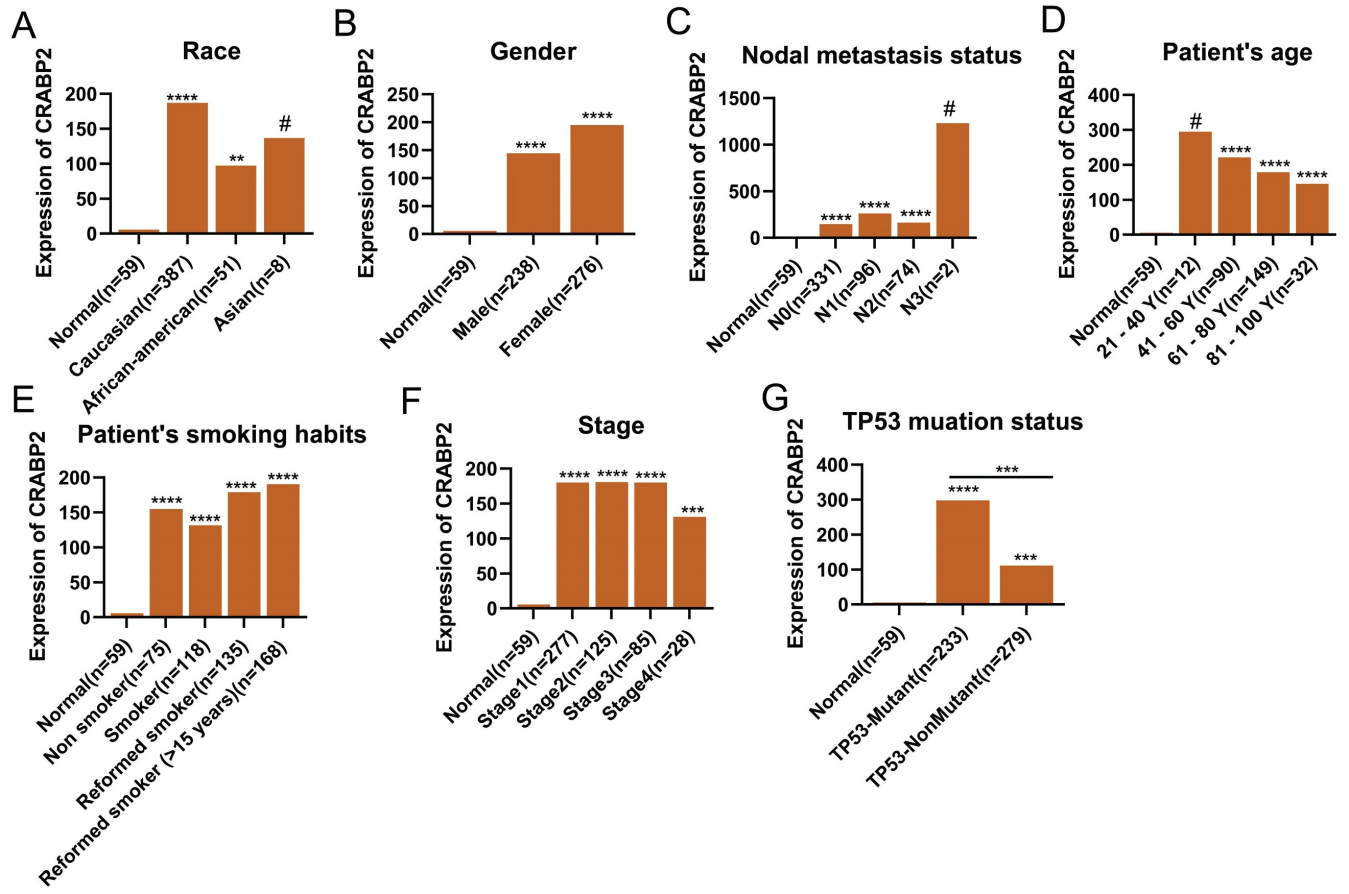
The difference between the expression of CRABP2 and ethnic populations in LUAD. LUAD: lung adenocarcinoma; ^#^*p*>0.05, ^*^p<0.05, ^**^p<0.01, ^***^p<0.001, ^****^p<0.0001, asterisks (*) stand for significance levels.

In addition, we explored the relationship between the expression of CRABP2 in LUAD and different clinical characteristic parameters of LUAD patients (Figure 3). Compared with normal people, CRABP2 shows significant differences with Caucasian and African-American people (Figure 3A). However, there were no significant differences among Asian groups, possibly due to the small sample size (Figure 3A). Similarly, compared with normal people, people of different Genders, Nodal metastasis status, Smoking habit and Stage all show significant differences, but there is no significant difference among all subgroups (Figure 3B-F). Interestingly, the expression of CRABP2 was significantly different between LUAN patients with TP53 mutation and LUAD patients without TP53 mutation (Figure 3G).

### Higher CRABP2 expression in LUAD patients predicts poor prognosis

**Figure 4:**
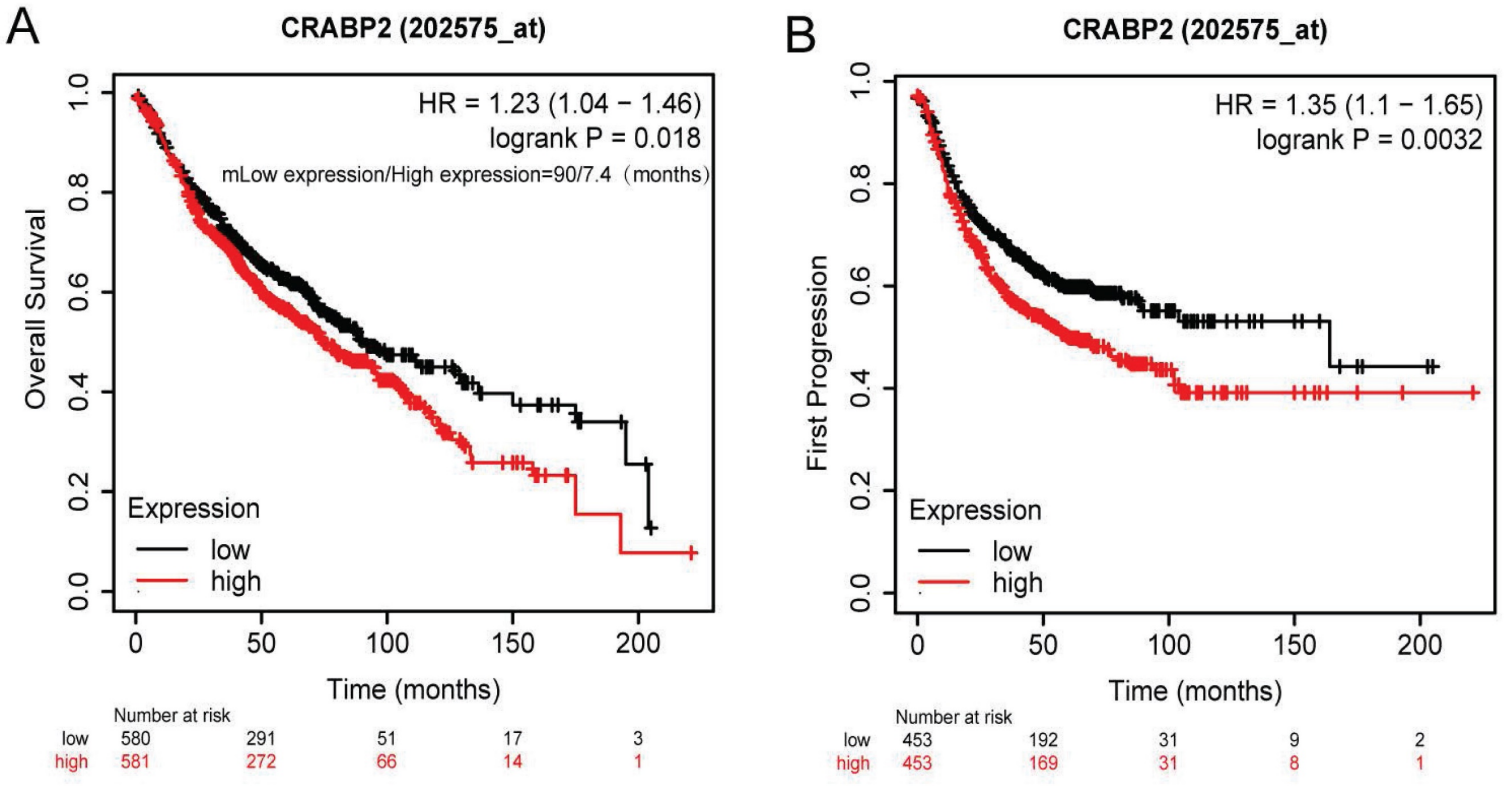
Survival of patients of CRABP2 through Kaplan-Meier plotter online analysis tool. (A) Relationship between CRABP2 expression and overall survival in LUAD patients. (B) The First Progression and Post Progression Survival analyses of CRABP2. LUAD: lung adenocarcinoma; GEPIA2: Gene Expression Profiling Interactive Analysis 2. HR: Hazard Ratio; *p*<0.05 was used to assess differences.

The results from our survival analysis showed that groups exhibiting CRABP2 overexpression had a reduced OS (Overall Survival) (95% CI: 1.04-1.46, HR: 1.23, *p*=0.018; Figure 4A). Specifically, the median OS for patients with low CRABP2 expression was 90 months, whereas for those with high expression, it was only 7.4 months. Additionally, the group with overexpressed CRABP2 also experienced a shorter First Progression (FP) time (*p*=0.0032, HR=1.35, 95% CI: 1.10-1.65; Figure 4B).

### High expression of CRABP2 in LUAD may potentially serve as a diagnostic and prognostic biomarker

As demonstrated above, the levels of CRABP2 expression in plasma are notably elevated in patients with early-stage LUAD as compared to healthy controls (Figure 1A). Additionally, the ROC curve analysis indicated that the plasma CRABP2 level distinguished LUAD patients in the TNM stage IA group from controls with an AUC of 0.839, exhibiting high specificity (Figure 1B). Furthermore, an investigation into the expression profile of CRABP2 in LUAD tumor tissues versus normal lung tissues revealed patterns consistent with those observed in plasma (Figure 2). Moreover, our survival analysis indicated that elevated CRABP2 expression in LUAD is predictive of poorer survival outcomes, affecting both OS and FP (Figure 4). Collectively, these findings suggest that the overexpression of CRABP2 in early-stage LUAD may potentially serve as a valuable biomarker.

### Enrichment analysis of the molecular function of CRABP2 in LUAD

**Figure 5:**
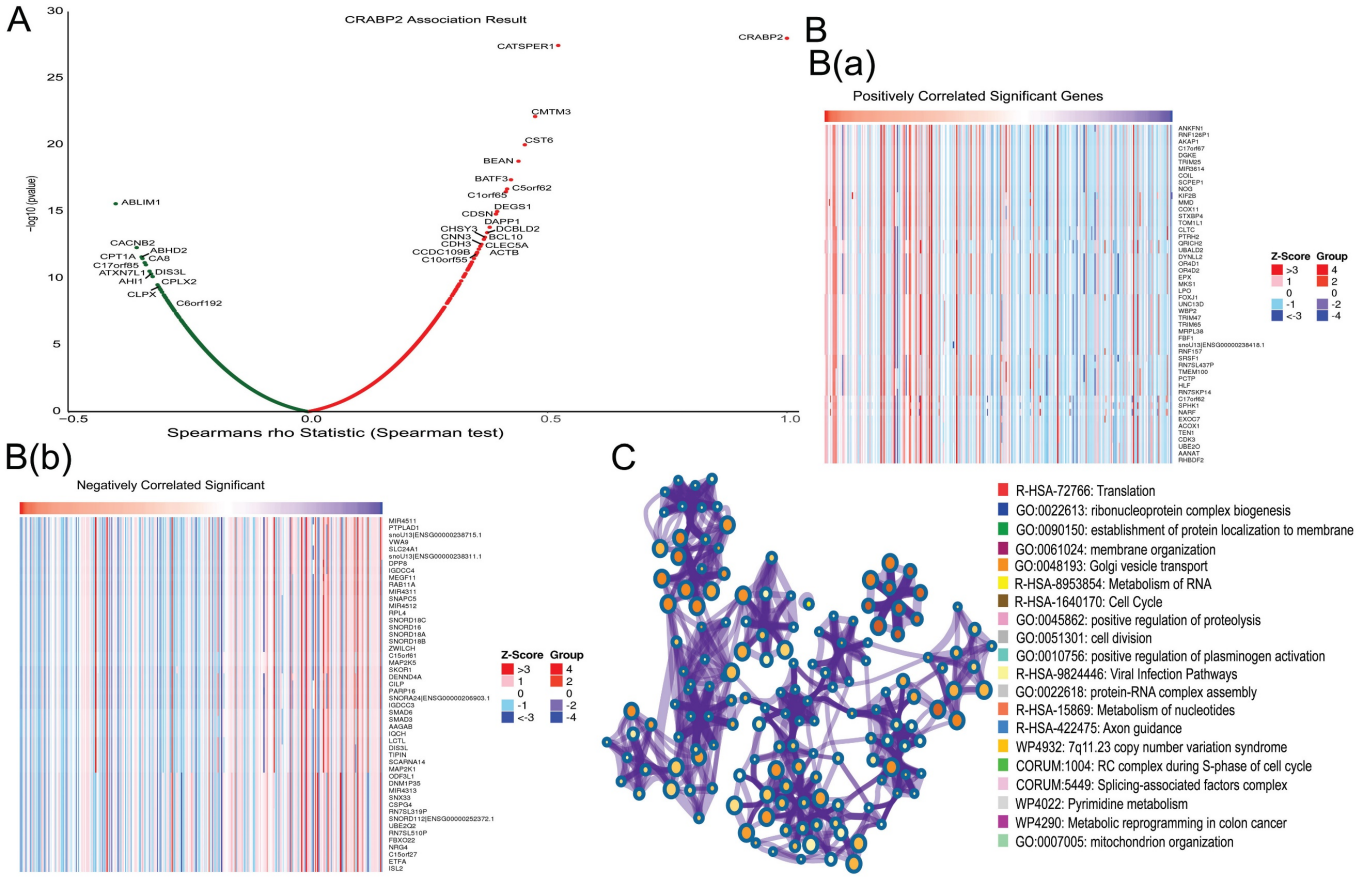
Functional enrichment analysis of CRABP2 in the development of LUAD. (A)The global CRABP2 highly co-expressed genes identified by the Spearman test in LUAD (LinkedOmics). Red and green dots represent positively and negatively significantly correlated genes with CRABP2, respectively. (B and C) Heatmaps showing the top 50 genes positively and negatively correlated with CRABP2 in LUAD (LinkedOmics).

We utilized the LinkFinder module in LinkedOmics to explore the co-expression patterns of CRABP2. As illustrated in Figure 5A, 13207 genes were found to be correlated with CRABP2 in LUAD, consisting of 5371 genes with a positive correlation and 7836 genes with a negative correlation (*p*<0.05). The top 50 genes exhibiting significant positive and negative correlations with CRABP2 are visually represented through heat maps in Figure 5B. Furthermore, an enrichment analysis revealed that the molecular functions of CRABP2 co-expressed genes in LUAD primarily involve Translation, ribonucleoprotein complex biogenesis, establishment of protein localization to the membrane, Golgi vesicle transport, RNA Metabolism, Cell Cycle, protein-RNA complex assembly, nucleotide Metabolism, positive regulation of proteolysis, mitochondrion organization, among others (Figure 5C).

Therefore, we believe that CRABP2 has been found to potentially regulate cell cycle progression, apoptosis, and mitochondrial function, all of which contribute to the progression of LUAD, thereby affecting the prognosis of LUAD patients.

### CRABP2 downregulation inhibits lung adenocarcinoma cell proliferation and arrests the G2/M phase, leading to increased cell apoptosis

**Figure 6:**
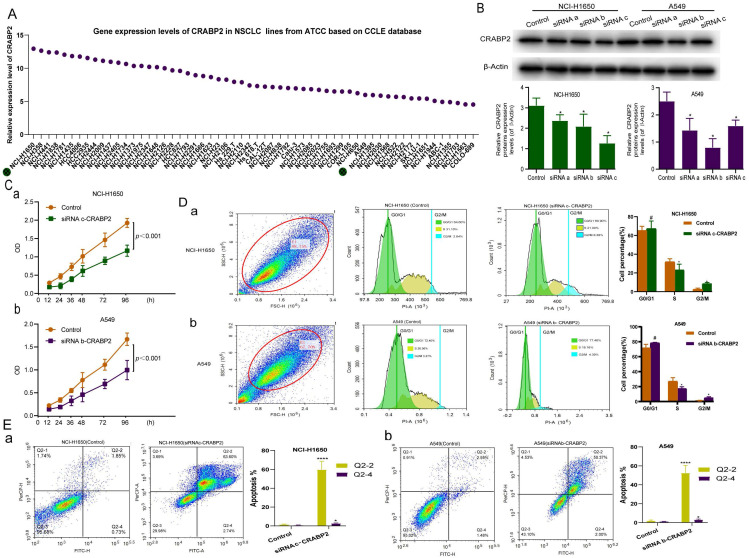
*In vitro* experiments to explore the molecular mechanism of CRABP2 in NSCLC. (A) Gene expression levels of CRABP2 in common human LUAD cell lines from the Cancer Cell Line Encyclopedia database. (B) Western blotting for analysis of the expression of CRABP2 after siRNA knockdown. (C) Effect of CRABP2 siRNA knockdown on the proliferation of NCI-H1650 and A549 cells by Cell Counting Kit 8 assay. (D-E) Effect of CRABP2 siRNA knockdown on NCI-H1650 and A549 cell cycle and cell apoptosis by flow cytometry. FDR: false discovery rate; LUAD: Lung adenocarcinoma. NSCLC: Non-Small Cell Lung Cancer; ATCC: American Type Culture Collection; CCLE: Cancer Cell Line Encyclopedia. ^#^*p*>0.05, ^*^p<0.05, ^**^p<0.01, ^***^p<0.001, ^****^p<0.0001, asterisks (*) stand for significance levels. *p*<0.05 was used to assess differences.

Based on the previous enrichment analysis of molecular function (Figure 5), we hypothesized that CRABP2 might facilitate LUAD progression by influencing cell cycle and apoptosis. To validate this hypothesis, we conducted *in vitro* experiments. To identify appropriate LUAD cell lines for siRNA-CRABP2 construction, we retrieved CRABP2 expression data for common human LUAD cell lines from the Cancer Cell Line Encyclopedia (CCLE) database hosted by the ATCC cell line bank. Our analysis revealed that NCI-H1650 and NCI-H358 cell lines exhibited the highest expression levels of CRABP2 (Figure 6A). Conversely, NCI-H1563 and COLO-699 cells demonstrated the lowest expression (Figure 6A). Taking availability into account and considering the availability, we selected NCI-H1650 and A549 as our experimental cell lines for this study.

To explore the role of CRABP2 in LUAD progression, we performed siRNA (siCRABP2) knockdown of CRABP2 in A549 and NCI-H1650 cells. Western blot analysis (Figure 6B) demonstrated a significant reduction in CRABP2 protein expression levels in both A549 and NCI-H1650 cells upon CRABP2 knockdown (*p* < 0.05).

Following the transfection of A549 and NCI-H1650 cells with siRNA-CRABP2 and subsequent culturing for 24 hours, we employed the CCK-8 assay to assess the impact of CRABP2 on the proliferation of these LUAD cell lines. The results indicated a notable decrease in cell activity from 12 to 96 hours after transfection with siCRABP2 (Figure 6C).

The functional enrichment analysis of CRABP2 in LUAD (Figure 5) implies that CRABP2 may regulate multiple cellular processes, including cell cycle progression, apoptosis, mitochondrial function, and other mechanisms, thereby promoting LUAD development and influencing the prognosis of LUAD patients. To assess the effects of CRABP2 downregulation on cell cycle and apoptosis, we employed flow cytometry in NCI-H1650 and A549 cells (Figure 6D-E). Upon transfection with siCRABP2, the percentage of NCI-H1650 cells in the G2/M phase rose from 2.84% to 8.28% (*p*<0.05), while the S phase cells decreased from 31.10% to 21.00% (*p*<0.05). Simultaneously, the G0/G1 phase cells increased marginally from 64.60% to 69.90% [Figure 6D(a)]. Similarly, in A549 cells, siCRABP2 transfection led to an increase in G2/M phase cells from 3.27% to 4.39% (*p*<0.05), a decrease in S phase cells from 26.56% to 18.16% (*p*<0.05), and a slight decrease in G0/G1 phase cells from 72.40% to 77.46% [Figure 6D(b)]. These findings suggest that downregulation of CRABP2 significantly induces G2/M phase arrest in both cell lines. Furthermore, treatment of NCI-H1650 and A549 cells with siCRABP2 resulted in a notable increase in early and late apoptotic cell ratios (Figure 6E). This indicates that CRABP2 plays a role in inhibiting apoptosis in NCI-H1650 and A549 cells.

### Analysis of CRABP2 expression and immune cell infiltration

**Figure 7:**
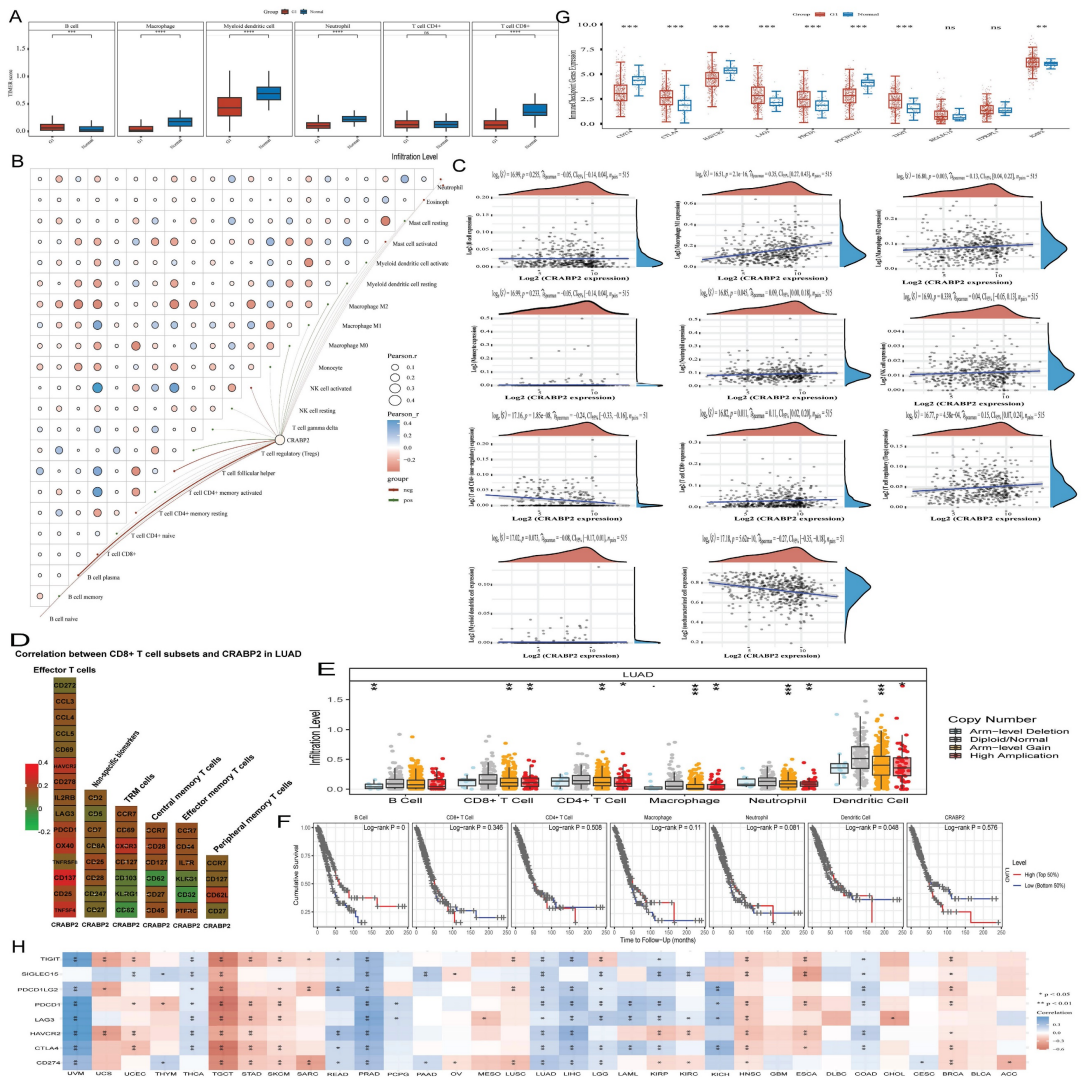
Correlation between the expression of CRABP2 and immune infiltration. (A) Effect of CRABP2 on the level of immune-related cell infiltration in LUAD through TIMER database; (B-C) EPIC immune (B) and CIBERSORTC immune (C) correlations between CRABP2 expression and immune score with Spearman. (D)The heat map of the correlation between the surface markers of different types of CD8^+^T cells and the expression of CRABP2. (E) Comparison of tumor infiltration levels among tumors with different somatic copy number alterations for CRABP2 by SCNA module based on TIMER database. (F) Survival difference between CRABP2 expression levels and immune cells using the Kaplan-Meier curve based on the TIMER database. (G) Relationship between the expression of CRABP2 and Immune-checkpoint-related gene in LUAD. (H) Correlation between CRABP2 and immune checkpoint genes in pan-cancer. *p < 0.05, **p < 0.01, ***p < 0.001, asterisks (*) stand for significance levels. Different colors represent the changes of correlation coefficients. *p*<0.05 was used to assess differences.

Moreover, CRABP2 expression and immunoreactivity score of immune cells including B cells, CD4^+^ T cells, CD8^+^ T cells, neutrophils, dendritic cells, and macrophages were investigated in LUAD using TIMER database. Compared to normal lung tissue, high expression of CRABP2 causes an increase in the proportion of B cells (Figure 7A). While, low expression of CRABP2 causes an increased proportion of CD8^+^T cell, Macrophage, Myeloid dendritic cell and Neutrophil (Figure 7A). It indicates that the low expression of CRABP2 will promote the recruitment of immune effector cells and reduce the proportion of immunosuppressive cells, thus playing a role in tumor inhibition in LUAD.

To verify the significance of CRABP2 in the tumor immune microenvironment, we used the CIBERSORT algorithm to evaluate the presence of 22 distinct immune cell types in LUAD samples (Figure 7B). Our findings revealed a notable correlation between CRABP2 expression and the immune scores of various cell types, including B cell memory, T cell CD4^+^ memory activated, T cell CD4^+^ memory resting, T cell regulatory (Tregs), T cell follicular helper, T cell gamma delta, NK cell activated, NK cell resting, Macrophage M0, Macrophage M1, Macrophage M2, Monocyte, Myeloid dendritic cell activated, Myeloid dendritic cell resting, Mast cell resting, Mast cell activated, and Eosinophil (Figure 7B, *p*<0.05). Furthermore, we conducted a Spearman correlation analysis between CRABP2 expression and immune scores using QUANTISEQ in LUAD samples (Figure 7C), which yielded consistent results with our initial findings.

The correlation between CRABP2 and immune infiltration cell marker genes in LUAD was investigated using both GEPIA2 and TIMER databases (Table 3). The results obtained from these two databases consistently suggest that CRABP2 expression is strongly linked to various immunological signatures, including general T cells, monocytes, CD8^+^ T cells, M1 and M2 macrophages, tumor-infiltrating lymphocytes (TILs), neutrophils, Th1 and Th2 cells, dendritic cells, regulatory T cells (Tregs), as well as exhausted T cells.

In addition, we analyzed the heat map of the correlation between the surface markers of different types of CD8^+^T cells and the expression of CRABP2(Figure 7D). We found that the expression of CRABP2 was significantly correlated with most of the surface markers of different types of CD8^+^T cells, including effector memory T cells, peripheral memory T cells, central memory T cells, TRM cells (tissue-resident memory T cells), effector T cells and non-specific biomarkers (Figure 7D).

Previous research has established that the tumor microenvironment is composed not only of tumor cells but also includes fibroblasts, stromal cells, and immune cells 30,31. Therefore, we evaluated the tumor infiltration levels of CD8^+^ T cells, CD4^+^ T cells, neutrophils, and macrophages in LUAD samples with varying somatic copy number alterations of CRABP2 (Figure 7E). Our findings further support a correlation between CRABP2 and immune cells within the tumor microenvironment. Alterations in CRABP2 expression or copy number can influence the tumor microenvironment, potentially playing a significant role in the onset, metastasis, and immune response of LUAD.

Next, we conducted a Kaplan-Meier survival analysis using data from the TIMER database to further investigate the survival differences between CRABP2 expression levels and immune cell infiltration. Our results revealed significant associations between B cell infiltration (*p* = 0) and dendritic cell infiltration (*p* = 0.048) with the prognosis of LUAD (Figure 7F).

In conclusion, CRABP2 can regulate the occurrence and development of cancer by regulating the LUAD tumor immune microenvironment. The high expression of CRABP2 will inhibit the recruitment of immune effector cells and promote the proportion of immuno-suppressive cells, thus promoting the progression of LUAD.

### The expression of CRABP2 in LUAD is closely related to immune checkpoint

The expression of CRABP2 in LUAD has been analyzed in relation to immune checkpoints, we found that the low expression of CRABP2 may enhance the expression of CD274(PD-L1), HAVCR2 and PDCD1LG2(PD-L2) in LUAD (Figure 7G). While, the high expression of CRABP2 may enhance the expression of CTLA4, LAG3, PDCD1(PD-1), TIGIT and IGSF8 in LUAD (Figure 7G). Therefore, we thought that patients with high expression of CRABP2 in LUAD may have suboptimal efficacy when treated with inhibitors targeting CD274, HAVCR2, and PDCD1LG2, whereas they may experience better efficacy with inhibitors targeting CTLA4, LAG3, PDCD1, TIGIT, and IGSF8.

At the same time, we analyzed the relationship between CRABP2 expression and immune checkpoints in pan-cancer, we found that CRABP2 expression exhibits a significant association with all eight immune checkpoint genes (CD274, PDCD1, CTLA4, PDCD1LG2, HAVCR2, SIGLEC15, LAG3, and TIGIT) in testicular germ cell tumor (TGCT), prostate adenocarcinoma (PRAD), and lung adenocarcinoma (LUAD) (Figure 7H). Similarly, in uveal melanoma (UVM) and liver hepatocellular carcinoma (LIHC), CRABP2 expression is significantly correlated with seven of these immune checkpoint genes (LAG3, CTLA4, PDCD1LG2, HAVCR2, PDCD1, CD274, and TIGIT). Overall, CRABP2 expression significantly relates to most of these eight immune checkpoint genes (Figure 7H, *p*<0.05 & *p*<0.01).

However, in certain cancer types, such as ovarian cancer (OV), pheochromocytoma and paraganglioma (PCPG), pancreatic adenocarcinoma (PAAD), mesothelioma (MESO), glioblastoma multiforme (GBM), diffuse large B-cell lymphoma (DLBC), bladder urothelial carcinoma (BLCA), cervical squamous cell carcinoma and endocervical adenocarcinoma (CESC), and adrenocortical carcinoma (ACC), especially in GBM, DLBC, and BLCA, there is no significant correlation between CRABP2 expression and most immune checkpoint genes. These results imply that high CRABP2 expression is linked to the formation of the tumor immune microenvironment in various cancers. Consequently, many cancer patients with elevated CRABP2 expression, such as those with TGCT, PRAD, LUAD, UVM, LIHC, thyroid carcinoma (THCA), stomach adenocarcinoma (STAD), skin cutaneous melanoma (SKCM), brain lower grade glioma (LGG), kidney renal papillary cell carcinoma (KIRP), head and neck squamous cell carcinoma (HNSC), kidney chromophobe (KICH), esophageal carcinoma (ESCA), colon adenocarcinoma (COAD), and breast invasive carcinoma (BRCA), may benefit from certain immune checkpoint inhibitor therapy. Conversely, patients with high CRABP2 expression in GBM, DLBC, and BLCA may not respond favorably to such therapy. Based on these observations, we postulate that CRABP2 expression could potentially serve as a biomarker for predicting immunotherapy efficacy in multiple cancers, thereby guiding anti-tumor immunotherapy strategies.

## Discussion

As an intracellular lipid binding protein, CRABP2 is associated with retinoic acid and is thought to regulate the signaling of retinoic acid in cells. Furthermore, elevated CRABP2 expression has been linked to a poorer prognosis and more advanced stages of endometrial cancer (EC), making it a valuable biomarker for identifying high-risk EC cases 32.

Based on the analysis of 640 LUAD patients and 640 matched healthy control plasma samples collected at Lishui Central Hospital between June 2018 and June 2023, this study revealed that patients with early-stage (stage IA) LUAD had significantly higher plasma CRABP2 levels compared to the control group. The predictive capability of CRABP2 expression in early LUAD plasma was analyzed using the ROC curve, revealing that CRABP2 exhibits a certain degree of accuracy in predicting early LUAD. Furthermore, the analysis of differential expression between LUAD tissue and adjacent normal lung tissues corroborates these findings, both at the mRNA and protein expression levels. We have further discovered that high CRABP2 expression in LUAD patients indicates a poorer prognosis, implying that modifying CRABP2 expression levels could potentially enhance the prognosis of LUAD patients (Figure 4). As such, CRABP2 may serve as a promising diagnostic and prognostic marker for LUAD.

CRABP2 has been found to facilitate the advancement of gastric cancer, as well as mitigate mitochondrial apoptosis and foster resistance to oxaliplatin in gastric cancer via DNA hydroxymethylation 33. Additionally, CRABP2 promotes the progression of thyroid cancer through the Integrin/FAK/AKT Pathway, making it a potential therapeutic target in the treatment of thyroid cancer 34. CRABP2 enhances the methylation of TRIM16 by elevating EZH2 expression, subsequently expediting the epithelial-mesenchymal transition in serous ovarian cancer cells 35. Moreover, CRABP2 stimulates the proliferation of dermal papilla cells via the Wnt/β-Catenin signaling pathway 2. CRABP2 plays a significant role in advancing glioblastoma and serves as a predictor of the effectiveness of all-trans retinoic acid in treating malignant gliomas 18. Notably, patients with esophageal squamous cell carcinoma (ESCC) who exhibit higher CRABP2 expression have a significantly longer overall survival compared to those with lower CRABP2 expression 36. In our study, we observed that CRABP2 expression was significantly elevated in the plasma of early-stage (IA) LUAD patients compared to healthy controls. Additionally, it was also notably increased in both intermediate and advanced-stage LUAD patients' tumor tissues. High CRABP2 expression in LUAD is indicative of a poorer prognosis for LUAD patients. Furthermore, CRABP2 has the ability to advance the progression of LUAD by facilitating the G2/M phase transition of LUAD tumor cells and suppressing the apoptosis of LUAD cells. Based on our findings, we believe that CRABP2 can serve as a novel biomarker for the diagnosis and prognosis of LUAD.

CRABP2 expression is closely associated with immune cell infiltration in cutaneous melanoma 37. The immune infiltration of tumor cells in LUAD is associated with lymph node metastasis and patient prognosis 38. Previous studies have found that DCs, macrophages, CD4^+^ T cells, and dendritic cells are important immune cells in the body, playing a wide range of anti-tumor effects 39,40. The expression of CRABP2 may be related to the heterogeneity of the tumor immune microenvironment, which could affect the response to immunotherapy 41. We found CRABP2 can regulate the occurrence and development of cancer by regulating the LUAD tumor immune microenvironment. The high expression of CRABP2 will inhibit the recruitment of immune effector cells and promote the proportion of immuno-suppressive cells, thus promoting the progression of LUAD. The findings suggest that the low expression of CRABP2 will promote the recruitment of immune effector cells and reduce the proportion of immunosuppressive cells, thus playing a role in tumor inhibition in LUAD. We thought that high expression of CRABP2 in LUAD patients may trigger an antitumor immune response, indicating that CRABP2 plays a crucial role in the immunomodulation of LUAD. This necessitates further experimental validation to delve into how CRABP2 influences the immune microenvironment of LUAD. Understanding the mechanisms of immune evasion in LUAD involving CRABP2 and developing novel immunotherapeutic strategies holds significant potential importance. CRABP2 can be used as a biomarker to predict immune responses. However, more experiments are still needed to further validate this.

In recent years, immunotherapy for LUAD has developed rapidly. PD-1 combined chemotherapy is the standard treatment for advanced LUAD 42. However, real world study showed that lasting responses and good long-term outcomes were limited to a small subset of patients 43,44. In one study, a negative correlation was observed between CRABP2 and immune checkpoint molecules PD-1, PD-L1, and CTLA-4 across various types of tumors, including breast cancer, melanoma, gastric cancer, and testicular germ cell tumors. Furthermore, CRABP2 may function as a biomarker for predicting the effectiveness of PD-1 inhibitors in melanoma patients 45. We discovered that reduced expression of CRABP2 in LUAD may pregulate the expression of CD274(PD-L1), HAVCR2 and PDCD1LG2(PD-L2). Conversely, elevated expression of CRABP2 in LUAD may upregulate the expression of CTLA4, LAG3, PDCD1(PD-1), TIGIT and IGSF8. Furthermore, CRABP2 in LUAD appears to regulate the immune response within the tumor microenvironment by suppressing the expression of certain immune checkpoint molecules, namely CD274 (PD-L1), HAVCR2, and PDCD1LG2 (PD-L2), while promoting the expression of other immune checkpoint molecules, including CTLA4, LAG3, PDCD1 (PD-1), TIGIT, and IGSF8. This could potentially reduce immunosuppressive signals and strengthen antitumor immune responses. Further verification through additional experiments is necessary. We thought that patients with high expression of CRABP2 in LUAD may have suboptimal efficacy when treated with inhibitors targeting CD274, HAVCR2, and PDCD1LG2, whereas they may experience better efficacy with inhibitors targeting CTLA4, LAG3, PDCD1, TIGIT, and IGSF8. These associations will play a crucial role in estimating the potential benefits of immune checkpoint inhibitor therapy for these patients. Therefore, we postulate that CRABP2 could serve as an efficient biomarker for predicting the effectiveness of immunotherapy.

## Conclusion

In summary, our research has confirmed that CRABP2 expression is elevated in the plasma of patients with early-stage (IA) LUAD as well as in the tumor tissues of patients with intermediate and advanced-stage LUAD. Moreover, the expression level of CRABP2 correlates with the prognosis of LUAD patients. CRABP2 promotes the G2/M phase transition of LUAD tumor cells, inhibits apoptosis, and additionally plays a role in regulating the immune microenvironment. High expression of CRABP2 in LUAD inhibits the recruitment of immune effector cells and increases the proportion of immunosuppressive cells, thereby promoting tumor progression. Low expression of CRABP2 may upregulate the expression of CD274(PD-L1), HAVCR2 and PDCD1LG2(PD-L2) in LUAD. In contrast, the high expression of CRABP2 may upregulate the expression of CTLA4, LAG3, PDCD1(PD-1), TIGIT and IGSF8 in LUAD. Patients with LUAD who have high expression of CRABP2 may exhibit suboptimal response to inhibitors targeting CD274, HAVCR2, and PDCD1LG2, whereas they may experience better response to inhibitors targeting CTLA4, LAG3, PDCD1, TIGIT, and IGSF8.

CRABP2 can be regarded as a promising biomarker for the diagnosis of LUAD, prognosis prediction, and therapeutic intervention. Our recent studies have established a firm groundwork for the advancement of LUAD therapy and paved the way for its clinical application.

## Supplementary Material

Supplementary figure.

## Figures and Tables

**Table 1:** The baseline characteristics of patients with LUAD and control in this analysis.

Characteristics	LUAD (N= 640)	Control (N =640)	*p*-value
**Age (years)**			**<0.0001**
Median	56.1	46.9	
Range	18-84	22-87	
**Sex-N(%)**			**<0.0001**
Male	188(29.4)	339(53)	
Female	452(70.6)	301(47)	
**Smoking History(%)**			0.195
Yes	80(12.5)	97(15.2)	
No	560(87.5)	543(84.8)	
**Pack-year (months)**			
Range	4-150	0.75-180	
**Race**			NS
Asian	640	640	
Non-Asian	0	0	
**ECOG performance status score-%**			NS
0	634(99.1)	640(0)	
1	6(0.9)	0	
**Underlying disease**			
Diabetes-N(%)			**0.0021**
Yes	44(6.9)	77(12)	
No	596(93.1)	563(88)	
Hypertension-N(%)			**0.0008**
Yes	154(24.1)	105(16.4)	
No	486(75.9)	535(83.6)	
Ischemic heart disease-N(%)			0.3031
Yes	20(3.1)	28(4.4)	
No	420(96.9)	612(95.6)	
Cerebrovascular accident-N(%)			0.3851
Yes	14(2.2)	20(3.1)	
No	626(97.8)	420(96.9)	
Chronic renal failure-N(%)			>0.9999
Yes	9(1.4)	8(1.3)	
No	631(98.6)	632(98.8)	
Chronic liver disease-N(%)			**0.0001**
Yes	17(2.7)	48(7.5)	
No	623(97.3)	592(92.5)	
Chronic airway disease-N(%)			**0.0077**
Yes	17(2.7)	37(5.8)	
No	623(97.3)	603(94.2)	
History of other cancers-N(%)			**<0.0001**
Yes	56(8.8)	13(2.0)	
No	584(91.2)	627(98)	
AJCC stage-N(%)			
IA1	430(67.2)		
IA2	187(29.2)		
IA3	23(3.6)		

N=number; LUAD=non small cell lung cancer; *p*<0.05 was used to asses differences.

**Table 2:** The details of 7 GEO datasets in this study.

Dataset	LUAD(Stage) Number					Normal	Platforms
	IA	IB	II	III	IV		
GSE40275	2	5	0	4	0	43	GPL15974
GSE10072	5	17	21	12	3	49	GPL96
GSE31210	114	54	58	0	0	20	GPL570
GSE31908	9	8	8	5	0	20	GPL96
GSE32863	16	18	11	12	1	58	GPL6884
GSE116959	12	16	29	0	0	11	GPL17077

**Table 3:** Correlation analysis between CRABP2 and immune cell marker gene in TIMER and GEPIA database.

Description	Gene markers	TIMER		GEPIA2	
		R	*p*	R	*p*
CD8^+^ T cell	CD8A	0.11	**1.29e-02**	0.11	**0.019**
	CD8B	0.092	**3.75e-02**	0.083	0.069
T cell (general)	CD3D	0.155	**4.06e-04**	0.16	**0.00044**
	CD3E	0.124	**4.85e-03**	0.12	**0.0076**
	CD2	0.125	**4.52e-03**	0.12	**0.0062**
B cell	CD19	0.031	4.80e-01	-1.30e-06	1
	CD79A	0.055	2.10e-01	0.021	0.64
Monocyte	CD86	0.218	**6.38e-07**	0.23	**4.20e-07**
	CD115 (CSF1R)	0.191	**1.33e-05**	0.2	**7.90e-06**
TAM	CCL2	0.182	**3.24e-05**	0.18	**4.80e-05**
	CD68	0.105	**1.71e-02**	0.11	**0.02**
	IL10	0.119	**7.10e-03**	0.13	**0.0032**
M1 Macrophage	INOS (NOS2)	0.032	4.74e-01	0.028	0.54
	IRF5	0.327	**3.75e-14**	0.33	**9.50e-14**
	COX2(PTGS2)	0.12	**6.50e-03**	0.13	**0.0055**
M2 Macrophage	CD163	0.091	**3.82e-02**	0.14	**0.0027**
	VSIG4	0.135	**2.16e-03**	0.15	**0.00074**
	MS4A4A	0.14	**1.44e-03**	0.15	**0.0012**
Neutrophils	CD66b (CEACAM8)	0.071	1.08e-01	0.12	**0.0064**
	CD11b (ITGAM)	0.22	**4.79e-07**	0.23	**2.80e-07**
	CCR7	0.114	**9.46e-03**	0.12	**0.011**
Natural killer cell	KIR2DL1	0.041	3.55e-01	-0.061	0.18
	KIR2DL3	-0.037	4.01e-01	-0.021	0.64
	KIR2DL4	0.037	3.99e-01	0.029	0.53
	KIR3DL1	-0.043	3.32e-01	-0.042	0.36
	KIR3DL2	-0.052	2.36e-01	-0.056	0.22
	KIR3DL3	-0.021	6.34e-01	-0.02	0.66
	KIR2DS4	-0.041	3.51e-01	-0.052	0.26
Dendritic cell	HLA-DPB1	0.177	**5.23e-05**	0.2	**6.00e-06**
	HLA-DQB1	0.173	**7.63e-05**	0.16	**0.00062**
	HLA-DRA	0.228	**1.93e-07**	0.24	**1.00e-07**
	HLA-DPA1	0.21	**1.69e-06**	0.23	**4.00e-07**
	BDCA-1(CD1C)	0.192	**1.10e-05**	0.21	**2.10e-06**
	BDCA-4(NRP1)	0.265	**1.09e-09**	0.26	**4.80e-09**
	CD11 (ITGAX)	0.175	**6.41e-05**	0.18	**7.30e-05**
Th1	T-bet (TBX21)	0.104	**1.87e-02**	0.1	**0.022**
	STAT4	0.207	**2.05e-06**	0.19	**3.10e-05**
	STAT1	0.161	**2.55e-04**	0.17	**0.00024**
	IFN-γ (IFNG)	0.06	1.76e-01	0.046	0.31
	TNF-α (TNF)	0.169	**1.21e-04**	0.17	**0.00012**
Th2	GATA3	0.111	**1.21e-02**	0.11	**0.019**
	STAT6	-0.031	4.76e-01	-0.022	0.63
	STAT5A	0.143	**1.15e-03**	0.15	**0.00087**
	IL13	0.006	8.88e-01	-0.0014	0.98
Tfh	BCL6	0.07	1.10e-01	0.063	0.17
	IL21	0.077	**8.25e-02**	0.083	0.069
Th17	STAT3	-0.041	3.58e-01	-0.0058	0.9
	IL17A	-0.008	8.48e-01	-0.015	0.74
Treg	FOXP3	0.225	**2.55e-07**	0.23	**3.10e-07**
	CCR8	0.173	**7.84e-05**	0.17	**0.00013**
	STAT5B	0.046	3.02e-01	0.057	0.21
	TGFβ (TGFB1)	0.319	**1.75e-13**	0.35	**4.40e-15**
T cell exhaustion	PD-1 (PDCD1)	0.192	**1.14e-05**	0.19	**3.60e-05**
	CTLA4	0.186	**2.22e-05**	0.19	**3.50e-05**
	LAG3	0.107	**1.54e-02**	0.097	0.033
	TIM-3 (HAVCR2)	0.226	**2.28e-07**	0.24	**1.20e-07**
	GZMB	0.081	6.70e-02	0.062	0.17
	PDL1(CD274)	0.249	**9.64e-09**	0.28	**2.70e-10**

Bold values indicate *p*<0.05. *p*<0.05 was used to assess differences.
