# Supplementary material for: CRABP2 (Cellular Retinoic Acid Binding Protein 2D): A novel biomarker for the diagnosis and prognosis involved in immune infiltration of lung adenocarcinoma
Source: J Cancer. 2025 Feb 3;16(5):1631–46. doi: 10.7150/jca.96518 (PMC11843236; doi:10.7150/jca.96518)
Supplement: Supplementary file 1 — Supplementary figure. [file jcav16p1631s1.pdf]

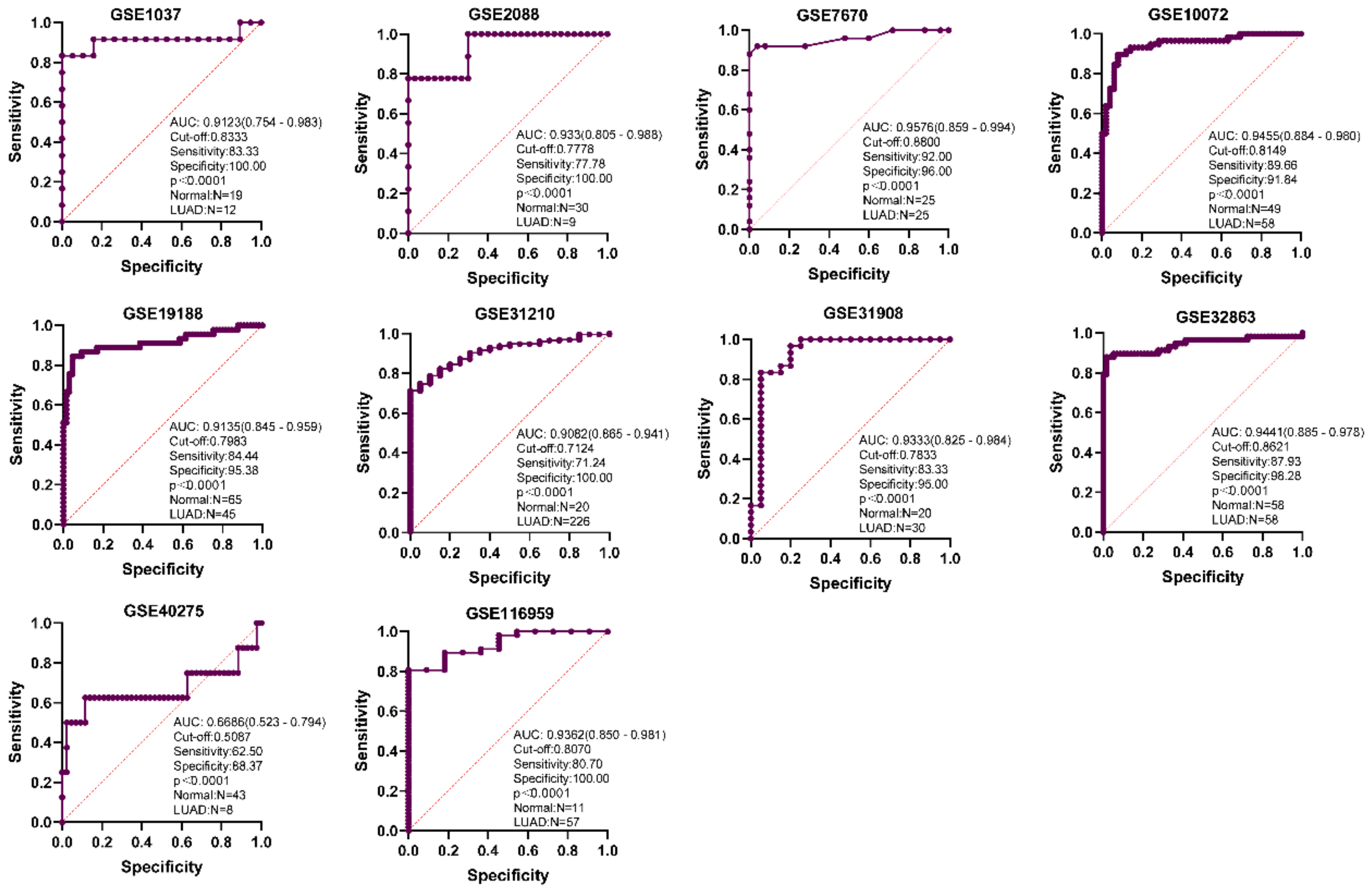

Supplementary materials S1 Receiver Operating Characteristic curve analysis of the diagnostic value of CRABP2 mRNA expression in Lung Adenocarcinoma.
